# Hybrid PET/Compton-camera imaging: an imager for the next generation

**DOI:** 10.1140/epjp/s13360-023-03805-9

**Published:** 2023-03-07

**Authors:** Gabriela Llosá, Magdalena Rafecas

**Affiliations:** 1 Instituto de Física Corpuscular (IFIC), CSIC-UV, Catedrático Beltrán, 2., 46980 Paterna, Valencia, Spain; 2 Institute of Medical Engineering (IMT), Universität zu Lübeck, Ratzeburger Allee 160, 23562 Lübeck, Germany

## Abstract

Compton cameras can offer advantages over gamma cameras for some applications, since they are well suited for multitracer imaging and for imaging high-energy radiotracers, such as those employed in radionuclide therapy. While in conventional clinical settings state-of-the-art Compton cameras cannot compete with well-established methods such as PET and SPECT, there are specific scenarios in which they can constitute an advantageous alternative. The combination of PET and Compton imaging can benefit from the improved resolution and sensitivity of current PET technology and, at the same time, overcome PET limitations in the use of multiple radiotracers. Such a system can provide simultaneous assessment of different radiotracers under identical conditions and reduce errors associated with physical factors that can change between acquisitions. Advances are being made both in instrumentation developments combining PET and Compton cameras for multimodal or three-gamma imaging systems, and in image reconstruction, addressing the challenges imposed by the combination of the two modalities or the new techniques. This review article summarizes the advances made in Compton cameras for medical imaging and their combination with PET.

## Introduction

“Scatter is the enemy”. This quotation is ascribed to Ed Hoffman, one of the great pioneers of positron emission tomograpy (PET). Although it mainly refers to Compton scatter in the patient, scatter in the detectors is also seen by many as a source of image degradation in PET. On the other hand, the potential of Compton scatter for imaging purposes has been long recognized. In fact, there are imaging techniques which fully rely on this kind of interaction, either in the patient (also known as *Compton-scattering imaging* [1]), or in the detectors. This document focuses on the latter. Among them, the most representative approach is the Compton camera (CC). Originally proposed for astrophysics, commercial Compton cameras are currently used, for example, in radiation protection and homeland security [2]. The potential of Compton cameras for medical applications was recognized long ago [3]. Initially, Compton cameras were considered as a possible alternative to clinical single-photon emission computed tomography (SPECT). Since conventional SPECT relies on gamma cameras with physical collimators, the sensitivity of this technology is intrinsically limited. On the contrary, Compton cameras rely on electronic collimation, so that a significant increase in sensitivity is possible.

In contrast to conventional PET or SPECT, where photoelectric absorption in the detectors is the goal, classical CC imaging requires that the emitted photons undergo a Compton interaction in a first detector (*scatterer*), and the resulting scattered photons also interact in a second detector (known as *absorber* when the scattered photon is expected to be fully absorbed). Using the kinematics of the Compton interaction, the original trajectory of a detected photon can be ideally constrained to the surface of a cone whose vertex corresponds to the first interaction; its axis is defined by the line connecting the first two interactions and it is assumed to be part of the scattered photon’s path. The cone aperture is derived from the scattering angle $\theta$, which is calculated as:

1 $$\begin{aligned} \cos \theta =1-\frac{m_{\rm e}c^2\Delta E_1}{E_0(E_0-\Delta E _1)}, \end{aligned}$$

where $m_{\rm e}$ denotes the electron mass, *c* the speed of light, $E_0$ the initial photon energy, and $\Delta E_1$ the energy deposited in the first interaction. The latter is assumed to correspond to the energy transferred from the original photon to the ejected electron in the Compton-scattering process. Next, tomographic reconstruction algorithms allow the spatial distribution of the emission origins to be estimated from the collection of cones (see Sect. 3.2). In electron tracking Compton cameras (ETCC), the direction of the scattered electron can be determined, thus reducing the uncertainty in the photon emission location and the complexity of the reconstruction.

The spatial resolution of Compton cameras improves with increase in photon energy. This is particularly advantageous to image radiotracers labeled with high-energy gamma emitters as well as therapeutic $\alpha$ and $\beta ^{-}$ emitters with additional high-energy $\gamma$ emission. On the other hand, such radionuclides challenge the performance of conventional gamma cameras. As high-energy photons can penetrate the collimator septa more easily, thicker septa walls are required, further reducing the detection efficiency. In addition, Compton cameras are better suited than gamma cameras for simultaneous imaging of multiple radiotracers, and a large field of view (FOV) can be imaged with small detectors. However, Compton cameras impose a higher technological challenge than gamma cameras, in particular due to their operation in time coincidence and the need of scatterers with high-energy resolution. With the low-energy gamma emitters employed in SPECT, current Compton cameras perform worse than gamma cameras and thus they cannot compete with this well-established technology. In addition, the image reconstruction process is more complex and the improvement in image quality resulting from a sensitivity enhancement is not as direct as in gamma cameras. These facts have prevented their expansion in medical applications.

Nevertheless, since the initial proposal for medical imaging, the interest in Compton cameras has been steadily growing, with an increase in number of research groups working on this field. Systems with different materials, in most cases developed for other applications, have been adapted and evaluated for medical imaging, including tests with small rodents and humans. In the last years, this technology has attracted renewed interest for medical applications in areas in which their advantages can be better exploited. Compton cameras are particularly well suited for prompt-gamma imaging (PGI) in hadron therapy, where photons in the MeV range are emitted [4], for dosimetry imaging in boron neutron capture therapy [5], as well as for certain radionuclide therapy treatments in which high-energy photon emission is a by-product [6]. In those cases, the photons can be employed to verify the drug distribution in the patient. Such poly-energetic photons are often characterized by energies higher than those commonly employed in SPECT, and thus, Compton cameras constitute a promising imaging option [6].

Another scenario where Compton cameras might outperform other techniques is multi-isotope imaging. The simultaneous administration of various tracers has great potential in many areas of brain, cardiac and oncologic imaging, since it allows exploration of different physiological and molecular functions under the same physiological and physical conditions [7]. Dual-isotope SPECT has been used since the nineties, for example, to differentiate between two diseases [8]. For this application, the selected collimator should match the photon energies of the two gamma emitters; therefore, the choice of the radiotracer pair is restricted by the collimator. Dual-isotope PET has been also proposed, although this modality is more demanding as the annihilation radiation alone does not allow the specific radiotracer to be identified. To cope with this, the combination of pure and non-pure positron emitters as well as the difference in half-lives and tracer kinetics can be exploited, [9–11]. Multitracer imaging is also being investigated with PET systems such as the J-PET scanner, with capability of tagging the events originating from various isotopes [12]. This scanner could be employed for dual tracer analysis (even with $\beta ^+$-$\gamma$ emitters see Sect. 4), such as ${}^{82}$Rb-Chloride, applied concurrently with ${}^{18}$F-fluorodeoxyglucose (${}^{18}$F-FDG) to enable simultaneous assessment of metabolic rate and perfusion, mainly in the cardiovascular system, or simultaneous applications of ${}^{44}$Sc-DOTATE and ${}^{18}$F-FDG for an early diagnostics of neuroendocrine and HER2-positive tumors.

Simultaneous PET/SPECT imaging has also been explored, following different approaches [13–18]. SPECT examinations made in addition to ${}^{18}$F-FDG PET scans can improve cancer diagnosis. In internal irradiation therapy with ${}^{131}$I for thyroid cancer, the comparison of ${}^{131}$I scintigraphy and ${}^{18}$F-FDG PET supports the decision to continue treatment [19]. In general, images in the two modalities are acquired independently, since the interference of the PET and SPECT systems can lead to sub-optimal performance. For instance, the presence of SPECT collimators reduces the PET sensitivity, and the interaction of 511-keV photons in the SPECT heads introduces an undesired background in the SPECT images. While some studies indicate the benefits of simultaneous imaging of PET and SPECT tracers [20], other studies show that this combination is challenging, and thus, sequential studies are preferred [21].

The advantages brought up by Compton cameras can be merged with the well established, high sensitivity PET imaging technique to profit from the benefits of each modality and make the most of the combination, or to enable new modalities such as three-gamma imaging. In this review article, we describe some of these concepts as well as recent achievements in this area.

Recently, two review articles on Compton cameras have been published focusing on different aspects, [22, 23], the latter including Compton cameras for non-medical applications.

## Positron emission tomography

PET is a well-established medical imaging technology which also relies on electronic collimation. This modality aims at detecting in coincidence the two 511-keV photons which originate from positron-electron annihilation. A PET scanner usually consists of rings of scintillation detectors, although open geometries have been also designed for certain applications, such as intra-operative imaging [24], online verification in particle therapy [25, 26], or imaging of specific organs, such as prostate, brain or breast [27]. PET/CT scanners from major commercial vendors are currently based on different types of SiPMs; the axial field of view has increased in most cases over 20 cm, and the time-of-flight (TOF) resolution is in the range of 214–380 ps [28]. Efforts are being invested in further improving the time resolution of the detectors to provide very accurate TOF information, with the final goal of achieving a coincidence time resolution (CTR) of about 10 picoseconds [29]. Latest advances in PET instrumentation have led to *total body PET*: commercial scanners with very large FOV allow the complete patient, or at least a large part of the body, to be scanned in one single-bed position achieving unprecedented sensitivity and allowing simultaneous multi-organ imaging [30, 31].

Although most conventional PET radionuclides are pure positron emitters, such as ${}^{18}$F, radionuclides with further decay modes have also become relevant in nuclear medicine, broadening the spectrum of PET applications but also posing new challenges to the instrumentation [32]. In particular, some of those radionuclides emit additional gamma-rays in the decay process, either in cascade with the positron as for ${}^{124}$I, or after some delay, as a result of electron capture (e.g., ${}^{89}$Zr). While some gamma-rays could be rejected using narrow energy and time windows, their contribution to the true PET signal (i.e., annihilation photons) cannot be completely eliminated. As a consequence, wrong information flows into the image, degrading contrast and adding background noise.

The spatial resolution of PET images is intrinsically limited by the range of the positron, which depends on the radionuclide. Whereas the resolution degradation is negligible for ${}^{18}$F, the most used PET radionuclide, it becomes a relevant source of image degradation in the case of ${}^{15}$O, ${}^{68}$Ga or ${}^{82}$Rb, to cite a few. In the last years, new developments in radionuclide targeted therapy offer new ground for PET and SPECT as imaging tools in theranostics (or theragnostics). In addition, there is growing interest in imaging nonstandard radionuclides characterized by very large positrons ranges (e.g., ${}^{72}$As). Although the degradation effects can be partially compensated for during the reconstruction process [33, 34], a certain loss of resolution cannot be completely eliminated. Not even the high TOF capabilities of modern scanners can overcome this problem, as TOF information serves to reduce the region where the annihilation occurred, but not where the positron came from. The limitation posed by positron range and the additional emission of single gamma-rays have motivated some groups to develop alternative detection concepts, which partly or fully rely on Compton kinematics and are referred here as to *hybrid PET/Compton camera* (see Sect. 4).

**Fig. 1:**
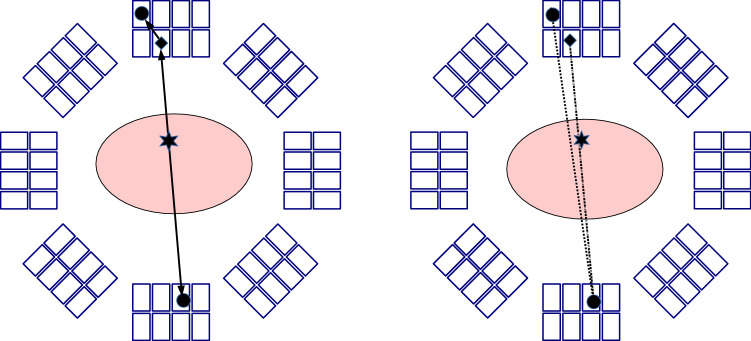
Illustration of inter-crystal scatter and a triple coincidence in a PET model with two layers of pixelated detectors. The black circle represents full absorption of a photon, whereas the rhombus denotes a Compton interaction and the star the origin of the two annihilation photons. Left: True trajectories of the photons. Right: Ambiguity in defining the true trajectory, as two LOR might be possible. The so-called “V”-trajectory is composed by the two possible LORs

Note that the term *Compton-PET* has been also employed in the literature to describe PET detectors able to identify and process Compton-scatter events [35], aka *inter-crystal* or *inter-detector scatter* events, or also to refer to PET systems in which Compton interactions in the detectors are considered as valid interactions [36]. The idea of exploiting inter-crystal events was long proposed as a possibility to increase the overall detection efficiency [37, 38]. This approach is mainly of interest for highly pixelated or multi-layer detectors. An inter-crystal scatter event commonly refers to an event in which an annihilation photon is detected through a Compton interaction followed by the photoabsorption of the subsequent scattered photon in a different crystal, as illustrated in Fig. 1, left; this implies that a coincidence event is composed by three or more detected single photons. In conventional PET, multiple coincidences are discarded, so that each coincidence event can be assigned a unique *line-of-response* (LOR), i.e., the line connecting the two detectors involved in the coincidence. The use of narrow energy windows further helps to suppress multiple coincidences; still, as Compton scatter is forward peaked, the interaction of the scattered photon and not the original one can fall within the energy window and thus be used to define a wrong LOR. To intentionally detect ICS events, large energy windows are used, as well as energy constraints. Nevertheless, due to the limited energy resolution of some detectors, the sequence of the two interactions related to the first annihilation photon cannot be easily determined, so that two or more possible LORs can be defined for that event, see Fig. 1, right. To disambiguate the LOR assignment, specific algorithms have been developed to identify the first interaction, either based on analytical considerations [39–41] or deep learning [35, 42]. Further options have been proposed for the so-called triple coincidences, i.e., the two original annihilation photons as well as the scattered photon, for instance using “V”-trajectories instead of LORs [43], or distributing the number of detected counts in a proportional manner [44].

The aforementioned approaches have shown that a significant increase in the sensitivity can be achieved without compromising the spatial resolution. However, the considered PET systems were primarily designed to detect coincidences of non-scattered annihilation photons, so that inter-crystal scatter was a by-product. In contrast, dedicated concepts have been proposed to actively produce and exploit inter-crystal scatter events, see Sect. 4.

## Compton cameras in medical imaging

Compton cameras have long been evaluated for medical applications given their promising advantages over collimated cameras: high sensitivity, large FOV, good separation of different isotopes and use of high-energy radiotracers with lower attenuation in the patient. Numerous Monte-Carlo simulations keep showing potential benefits [45–48] that are still hard to translate into clinical use, since the competition with a well-established and affordable technology such as SPECT is complex. In particular, many approaches have not yet reached sufficient maturity in terms of technology and software development, e.g., image reconstruction or corrections for attenuation and scatter. Nevertheless, Compton cameras continue their progress improving their performance. Different types of detectors have been used in their development, from solid-state detectors, which offer excellent energy and spatial resolution, to scintillators coupled to SiPMs that can facilitate their clinical application [49]. While initially many tests were carried out with detectors that were developed for other applications, systems employed in recent experiments start to face the requisites for medical use.

### CC developments

The application of Compton cameras to medical imaging was proposed by Todd et al. [3] and followed by Singh and Doria [50] who developed several prototypes for different purposes. Their first experimental prototype was made of a high-purity Germanium (HPGe) detector as scatterer and a NaI absorber and was able to image ${}^{\rm 99m}$Tc and ${}^{137}$Cs sources and 3D cylindrical test phantoms in later experiments [51]. In the following set of experiments, silicon detectors were selected as scatterer, given their low Doppler broadening, excellent energy and spatial resolution and simpler operation even at room temperature, not requiring external cooling. The first experiments with silicon detectors [52] were continued by the same and other groups [53–55].

Other solid-state detectors have also been employed. The first images of a living mouse were obtained with a Compton camera composed of two double-sided strip Ge detectors. In vivo 2D and 3D images were obtained injecting three radiotracers simultaneously to a mouse under anesthesia [56]. Si/CdTe Compton cameras have also been employed for multi-isotope imaging of small animals [57, 58] and even a human volunteer who was injected intravenously 30 MBq of ${}^{99m}$Tc-DMSA and 150 MBq of ${}^{18}$F-FDG simultaneously [59]. Such systems demonstrated in vivo multi-isotope imaging with a Compton camera, while the performance was still far from PET and SPECT and the systems were not suitable for being operated in a clinical environment.

SiPMs have enabled the use of scintillator detectors also as scatterers simplifying operation and lowering the cost of Compton cameras, while the energy resolution worsens with respect to solid-state detectors. GAGG is a cost-effective scintillator that has also allowed small animal imaging. In [60] a mouse was imaged after injection of three radiotracers, ${}^{{\rm 131}}$I, ${}^{{\rm 85}}$Sr, and ${}^{{\rm 65}}$Zn, showing concentration in their specific target organs. SiPMs have also been used in miniature detector designs, such a laparoscopic Compton camera of 11.8 mm diameter for radio-guided surgery with four layers of GAGG pixelated crystals [61].

Other detector types and configurations have also been explored for medical applications. An ETCC was tested with 204 keV, 582 keV and 835 keV photons from ${}^{{\rm 95m}}$Tc that could potentially replace ${}^{{\rm 99m}}$Tc, reducing scattering in the patient [62]. Liquid Xenon (LXe) Compton cameras [63], ETCC with micropixel gas chambers [64] or employing Timepix [65] have also been developed.

**Fig. 2:**
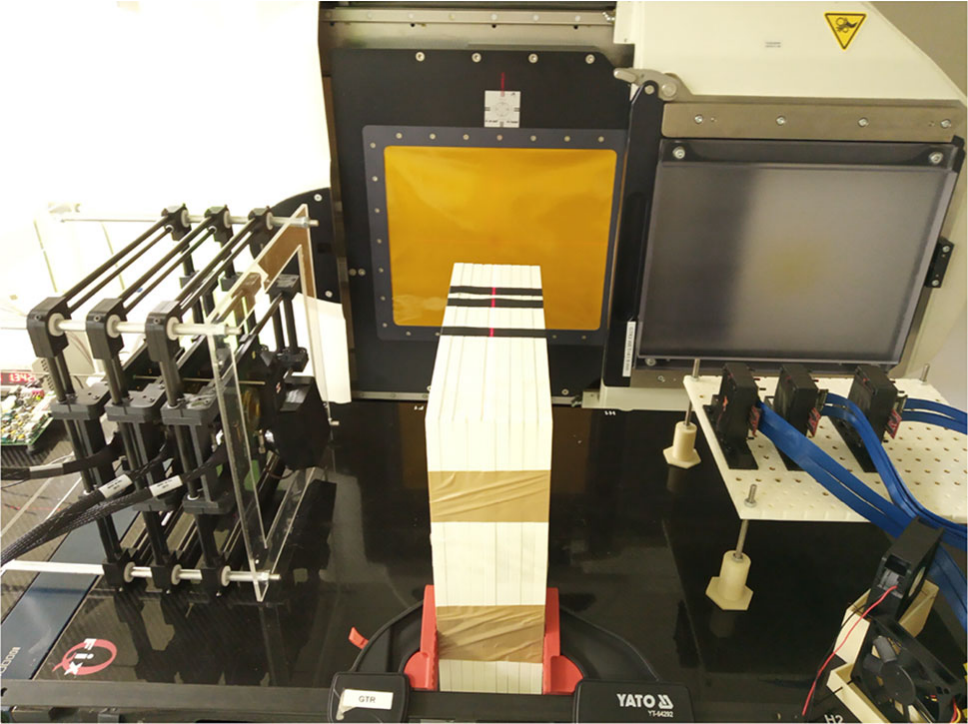
MACACOIII (left in the image), and MACACOp (right) Compton telescope prototypes being tested at the proton therapy center in Krakow

Compton cameras have also been proposed for monitoring treatment delivery in hadron therapy by imaging the photons (known as prompt gammas) emitted by the tissue upon irradiation [4]. Such photons have energies in the MeV range, higher than in diagnostic imaging. High count rate and particle background in the clinical treatment conditions impose challenging requisites to the systems. Two-stage and multi-stage Compton cameras with different types of detectors have been proposed, mostly based on scintillator or semiconductor detectors or a combination of both. Some of the systems have been able to detect Bragg peak shifts in proton beam experiments [66–69]. Figure 2 shows an image of the latest versions of MACACO Compton telescope prototypes, MACACOIII and MACACOp, being tested at the proton therapy center in Krakow.

Recently, Compton cameras are being tested for monitoring drug delivery in radionuclide therapy with alpha particles. The radionuclides employed (such as ${}^{{\rm 149}}$Tb, ${}^{{\rm 211}}$At, ${}^{{\rm 212}}$Bi, ${}^{{\rm 213}}$Bi, ${}^{{\rm 223}}$Ra, ${}^{{\rm 225}}$Ac, ${}^{{\rm 227}}$Th, among others) emit secondary photons that can be employed to image the drug distribution. Given their high energies, Compton cameras can offer a potentially better alternative [6, 47] to gamma cameras. Different systems are being tested, e.g., for imaging ${}^{{\rm 211}}$At [70], or combining a pinhole and a Compton camera to detect both X-rays and gamma-rays for imaging a mouse [71]. Imaging of ${}^{{\rm 223}}$Ra, widely used for treating bone metastases of prostate cancer in radionuclide therapy, has also been tested [72].

The development of Compton cameras has made significant progress and technological advances translate into a better performance. Tests show successful multitracer imaging ability and improving spatial resolution, currently in the order of few millimeters, that can be further enhanced. While gamma cameras constitute a well-established and cost-effective technology that can hardly be overcome, Compton cameras can offer alternatives in specific fields or dedicated systems in which their characteristics can be advantageous.

### Image reconstruction

The most appropriate procedure to create images from measured Compton camera data depends on the application. Simple and fast algorithms might be sufficient for “far-field” imaging (e.g., astrophysics) or radiating single point sources. In these cases, the reconstructed images are usually restricted to the projection onto the unit sphere.

**Fig. 3:**
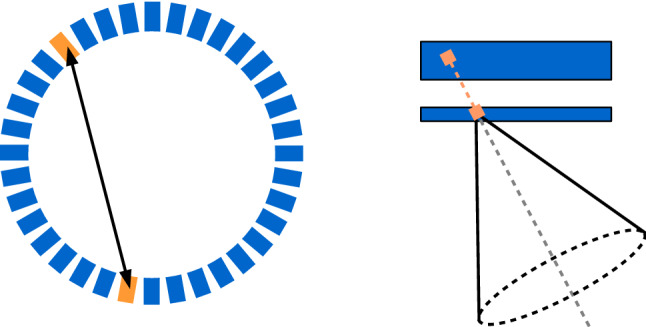
Schematic representation of the concept of line-of-response (LOR) in PET (left), and cone-of-response (COR) in a Compton camera (right)

For “near-field” applications, such as medical diagnostics or therapy monitoring, the image reconstruction problem becomes more complex. In most cases, the goal is to provide an estimate of a continuous 3D function, $f(\textbf{r})$ (with $\textbf{r} \in \mathbb {R}^3$), that either describes the radionuclide distribution within the body or, in the case of prompt-gamma imaging, the spatial distribution of the emission origins. Conceptually, the underlying inverse problem is very similar to other tomographic imaging modalities such as PET and SPECT. In fact, many reconstruction approaches originally developed for the latter have been extended to CC imaging. However, some important features of Compton cameras hinder a straightforward implementation of these algorithms. A very relevant difference concerns the spatial information provided by each measured event. In PET, the origin of a detected pair of photon is ideally constrained to the line connecting the two detectors in coincidence, the LOR. In Compton cameras, the region assigned to each measured event is, in the ideal case, the surface of a cone (*cone-of-response*, COR) (see Fig. 3). From this perspective, a PET coincidence event is more informative that a Compton camera event. Note that this statement does not apply to ETCCs, since in that case the origin of the Compton camera event could be constrained to a small segment of the cone. Another fundamental difference is the dimension of the measurement space: To characterize a PET event, only the coordinates of the two interactions are needed; in this way, the LOR is unambiguously defined. For pixelated PET detectors, the input can be reduced to a single index $i \in \mathbb {N}$ per event, with $1 \le i \le I$, so that dimension of the measurement space is simply *I*, the number of detector pairs in coincidence. This allows the measured data to be easily histogrammed, either according to the index *i* (*LOR histograms*), or using the parameters of the LOR (*sinograms*). Both methods can be mathematically expressed through the measurement vector $\mathbf {{y}}$, where ${y}_i$ corresponds to the number of events detected by the *i*th pair of detectors or in the *i*th sinogram bin. In contrast, the characterization of a Compton camera event through its related COR requires more parameters: vertex, axis and opening angle $\beta$ of the cone. Even if pixelated detectors are used (real or virtual), the dimension of the measurement space is much larger. If the finite energy binning is ignored, $\beta$ is a continuous variable and the corresponding dimension thus becomes infinite. On the other hand, discretization of $\beta$ or the other cone variables worsens the spatial resolution. These facts hinder the storage of Compton camera data in the form of histograms, so list-mode (LM) data are used instead.

In emission tomography, data completeness is essential to obtain artifact-free images. In PET, this is achieved using a full ring of detectors and minimizing the gaps between detectors. The combination of the numerous detector units in coincidence allows for sampling the field of view at many different angles. For Compton cameras, it is less obvious to identify how a complete and uniform sampling can be achieved. According to the few published works on this topic (e.g., [73]), extremely large absorbers would be needed, otherwise truncation artifacts might deteriorate the image. Alternatively, rotating the Compton camera [74] or using two or more Compton-camera heads at different angular positions [75] can also enlarge angular coverage and thus reduce truncation artifacts. Some compensation for data truncation can be reached using statistical iterative reconstruction methods in combination with very accurate models (see below). In any case, image quality is very sensitive to the size of the absorber, the distance between the camera and the patient, and the separation between absorber and scatterer, as these parameters contribute to determine the geometry of the cone surface which can be detected.

At present, statistical iterative approaches are the preferred option to reconstruct PET, CC or hybrid PET/CC data, while analytical reconstruction is seldom used. In the context of Compton-camera imaging, analytical algorithms were at first limited to reconstruct the image projected onto the unit sphere, e.g., using a spherical harmonics expansion of the measured data [76]. Significant advances have been made to provide an exact solution of the conical Radon transform [74, 77–79]. Analytical reconstruction techniques are attractive because of their speed, as they provide closed-form solutions to obtain the sought image. These exact formulas rely on an idealized description of the detection process, e.g., point-like detectors, no statistical fluctuations, complete set of projections, etc. Deviations from these assumptions create artifacts that might strongly distort the image, although some analytical approaches specifically addressing these issues have been also proposed [80, 81]. On the other hand, statistical reconstruction techniques can better cope with the intrinsic statistical fluctuations of emission data, measurement uncertainties and other physical phenomena which contribute to degrade the image. These techniques rely on a probabilistic description of the data, a model of the system response (*system matrix*) and a criterion to define the sought image (*objective function*). As statistical model for emission tomography, the Poisson probability distribution is considered the most appropriate one to describe the fluctuations of the measured data around their mean. For histogrammed data, the measurement process can be easily described by an algebraic set of equations, which in its compact form can be written as $\bar{\textbf{y}}= \textbf{A} \mathbf {{f}}$, where the vector $\textbf{f}$ describes the discretized unknown image. In radionuclide imaging, the vector element $f_j$ thus corresponds to the number of decays originating from image voxel *j* during the measurement or, equivalently, to the activity concentration. Vector $\bar{\textbf{y}}$ contains the expected number of detected events, i.e., $\bar{y}_i = E[{y}_i]$, thus reflecting the statistical nature of the emission and detection process. An element of matrix $\textbf{A}$, $a_{ij}$, corresponds to the probability that an event originating within image voxel *j* will be detected by the measurement element *i* (e.g., LOR index in PET). As $a_{ij}$ mainly depends on the characteristics of the imaging device, $\textbf{A}$ is often referred to as the *system matrix*. $\textbf{A}$ is a key element in statistical reconstruction as it links the measurement and image spaces through the forward- and backprojection operations, therefore it is also called *transition matrix*. A common criterion to define the unknown image in emission tomography is the *maximum likelihood* (ML), i.e., the sought distribution is the one which maximizes the likelihood function $L(\textbf{f})$, with $L(\textbf{f}) \equiv p[\textbf{y} \mid \bar{\textbf{y}}]$; the latter function is the joint probability distribution of the measurement, which is linked to the unknown image through the system matrix (see above). The likelihood function, as objective function, assesses how well a particular image describes the measured data. For Poisson-distributed data, the maximum of $L(\textbf{f})$, or equivalently the log-likelihood $l(\textbf{f})\equiv \log ~L(\textbf{f})$, is found in an iterative manner, being the *Maximum Likelihood Expectation Maximization* (MLEM) the most commonly used procedure. For histogrammed data, the *k*th iteration of MLEM is described by:

2 $$\begin{aligned} f_j^{(k+1)} = \frac{f_j^{(k)}}{s_j} \sum _{i=1}^{I} \frac{y_i}{\sum _{m=1}^J a_{im} f_m^{(k)} } a_{ij} ~~~~ {\rm with} ~~~~ {s_j} \equiv \sum _{i=1}^{I} a_{ij}. \end{aligned}$$

The elements ${s_j}$ express the detection sensitivity of the system to activity located in voxel *j*. Therefore, the corresponding vector ${\textbf {s}}$ is often termed *sensitivity* and has the same dimensions as the unknown image. Note that the denominator with the sum can be interpreted as $y_i^{(k)}$: this quantity represents the expected number of events to be measured by the detection element *i* if the unknown object were described by $\textbf{f}^{(k)}$, i.e., $\textbf{y}^{(k)} \equiv \textbf{A}\textbf{f}^(k)$. This operation is termed *forward-projection*. For list-mode data, MLEM is reformulated as LM-MLEM [82]:

3 $$\begin{aligned} f_j^{(k+1)} = \frac{f_j^{(k)}}{s_j} \sum _{i=1}^{N} \frac{1}{\sum _{m=1}^J a_{im} f_m^{(k)} } a_{ij}, \end{aligned}$$

where now the first sum is not carried out over all detection elements, such as the number of possible LOR in PET, but over *N*, the number of detected events. While the meaning of $s_j$ remains the same, $a_{ij}$ needs to be reinterpreted: For LM reconstruction, it corresponds to the probability that the *i*th measurement in the list originates from image element *j*. Hence, the index *i* does not define a fixed detection element, but it depends on the particular measurement.

**Fig. 4:**
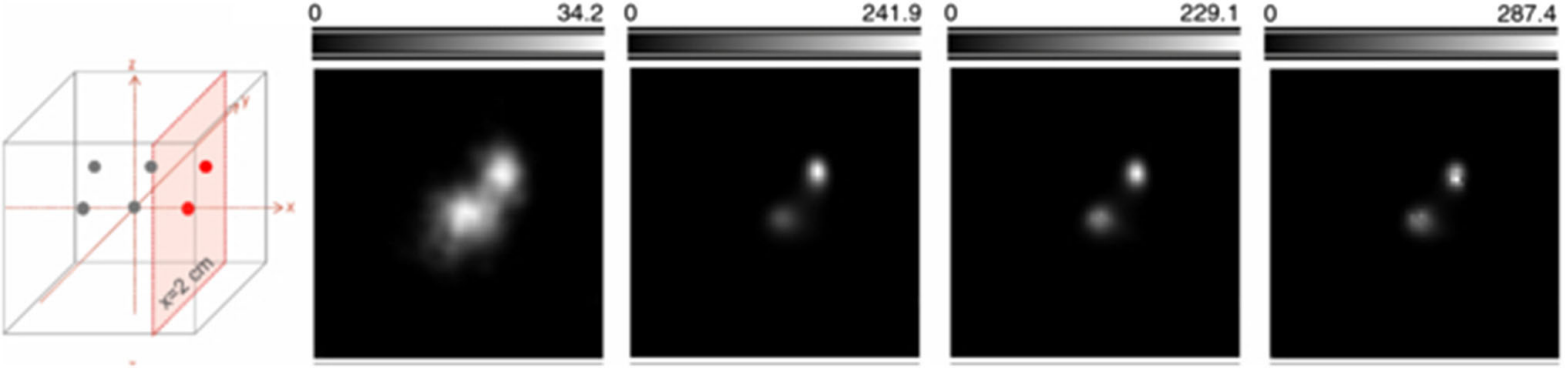
Effect of including a model of the PSF in the reconstruction. From left to right: Reconstructed plane depicting the simulated sources (parallel to the CC); plain LM-OSEM, and LM-OSEM with three different PSF models, namely a symmetric Gaussian kernel, an asymmetric Gaussian kernel and a general parametric function. Image from [83]. (Image reprinted with permission of $\copyright$ Institute of Physics and Engineering in Medicine. Reproduced by permission of IOP Publishing Ltd. All rights reserved)

LM-MLEM is particularly well suited when the number of detected events is much lower than the dimensions of the measurement space. This is precisely the case of Compton cameras. For this application, the elements $a_{ij}$ are usually computed for each measurement, mostly on the fly; in contrast, the elements of $\textbf{s}$ can be calculated off-line and stored on disk as, ideally, they do not depend of the measurement but only on the device. The quality of the reconstructed images strongly depends on the accuracy of $\textbf{A}$ and $\textbf{s}$ to describe all relevant phenomena involved in the detection process. In the first place, $a_{ij}$ accounts for the overlapping between the cone surface assigned to the *i*th event and the voxel *j*. Several approaches have been proposed to numerically compute this contribution, e.g., [84–87], for example, by sampling the COR with a finite number of generatrices and calculating the intersection between these lines and voxel *j*, using the Euclidean distance or the angular separation between the voxel and the COR surface, etc. If the finite size of the detectors and other measurement uncertainties are taken into account (e.g., limited energy resolution, Doppler effect, etc.), the concept of COR can be generalized to a *volume of response* (VOR), which represents the region in a 3D space where the origin of the event originates. Several approaches have been introduced to model the VORs into the reconstruction, see, for example [83, 88–92]. Among them, including a model of the point-spread-function (PSF) in image space as a convolution kernel is an extended option, see Fig. 4 .

**Fig. 5:**
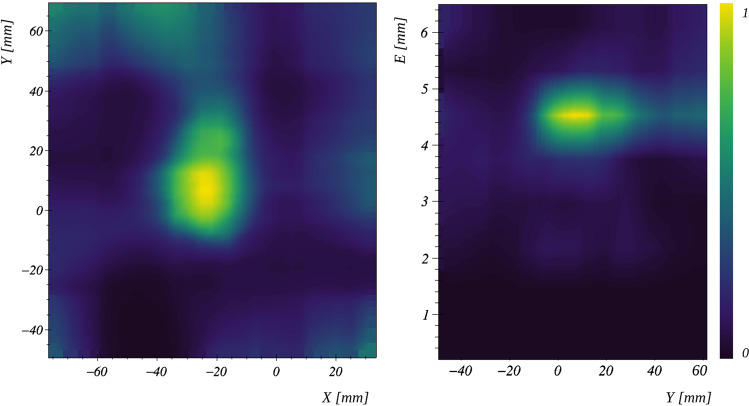
Spatial-spectral reconstruction of experimental data obtained with MACACO III at the proton therapy center in Krakow, with 90 keV protons impinging a RW3 phantom. Left: Spatial distribution of the reconstructed prompt-gamma distribution, obtained after projecting along the spectral dimension. The *Y*-axis is parallel to the direction of the proton beam. Right: Spectral-spatial distribution after projecting along the *X* direction, with photon energy peaking at 4.4 MeV. (Courtesy of J. Roser, Instituto de Física Corpuscular (IFIC, CSIC-UV), Valencia, Spain)

Ideally, the system matrix should also model the probabilities of attenuation and possible interaction in the detectors [82, 87, 93]. These probabilities do not only depend on the emission location and distance to and between the detectors, but also on the energy of the incoming radiation, $E_0$. In the case of radionuclide imaging, $E_0$ is known a priori, as opposed to PGI for range verification in particle therapy. In the latter scenario, the spectrum of the prompt-gamma radiation covers a quasi-continuous and large range, over 10 MeV. Under the assumption of photoelectric effect in the last detector, the unknown energy $E_0$ can be retrieved, but given the high energy of PG photons, full absorption is rare, so that the COR is calculated using a wrong estimation of $E_0$. Alternatively, three or more interactions could also allow for calculating $E_0$ [94]. To this aim, specific Compton camera concepts with more than one scatterer are needed, as well as specific modeling [95]. Still, this kind of events is much less frequent than coincidences made of two interaction. To exploit the more abundant although less informative two-interaction events, spectral–spatial reconstruction approaches have been proposed [96–100]. The idea consists in including the energy as a further unknown and reconstruct $E_0$ together with the spatial distribution. By projecting the reconstructed distribution over the spectral dimension, spatial images are obtained (see Fig. 5).

In this way, a more accurate, energy dependent modeling can be included into the reconstruction process but at the expense of increasing the complexity and computation time. In [101], the authors compare conventional spatial reconstruction and spatial-spectral reconstruction for polychromatic sources, showing that the latter method outperforms conventional reconstruction to a certain extent.

The flexibility of the ML formulation not only allows for extending the image space into the spectral dimension, but also for considering different detection combinations (“channels”). This idea was first exploited for a Compton-PET system (see [102] and Sect. 4) and was recently adapted to multi-scatterer Compton camera to take into account the various detector planes and their different sensitivities [103].

To accelerate the reconstruction, one possibility consists in grouping the data into subsets and splitting up the iteration process into so many sub-iterations as subsets. This is the essence behind the *ordered subset expectation maximization* (OSEM) algorithm, which has been also adapted for Compton camera data [85, 104, 105]. Originally, geometrical considerations were employed to create the subsets; for LM data, temporal subsets are better suited. In this case, the algorithm consists of two steps, where a sub-iteration through the events of the *l*th subset, $N_l$, is described by:

4 $$\begin{aligned} f_j^{(k, l+1)} = \frac{f_j^{(k, l)}}{s_j} \sum _{i\in N_l} \frac{1}{\sum _{m=1}^J a_{im} f_m^{(k,l)} } a_{ij}; \end{aligned}$$

next, the last iterate is updated, $f_j^{(k+1),0}:= f_j^{(k, L)}$, where *L* corresponds to the number of subsets. Note that OSEM with $L=1$ is LM-MLEM. OSEM is available as *mimrec* [106] within *Medium-Energy Gamma-ray Astronomy library* (MEGAlib), an open-source software specifically conceived for Compton cameras [107]. In general, the acceleration factor is proportional to *L*, but the image can become distorted if few events per subset or unbalanced subsets are used.

In case of low-count measurements, as PGI in particle therapy, images reconstructed with ML-based approaches might suffer from prohibitively high levels of statistical noise. Noise amplification is in fact a natural consequence of using the likelihood as objective function, as it forces the reconstructed image to fit the noisy measured data. To tackle this issue, several strategies are possible, such as smoothing the images after convergence, using larger voxels or alternative image basis functions, stopping the algorithm before convergence, or using some a-priori knowledge about the unknown distribution. The latter can be integrated in a Bayesian framework through the *Maximum-A-Posteri* (MAP) objective function. The goal of MAP is to find the image which maximizes the posterior probability function, $p[\textbf{f} \mid \textbf{y}]$, instead of the likelihood $p[\textbf{y}\mid \textbf{f}]$. The Bayes theorem and a logarithmic transformation reduce the optimization problem to maximize $l(\textbf{f}) + p[\textbf{f}]$, where $p[\textbf{f}]$ corresponds to the prior probability of a given image $\textbf{f}$. Now the key issue is to formulate the a-priori knowledge within $p[\textbf{f}]$. When the image is interpreted as Markov random field, the objective function becomes $l(\textbf{f}) - \beta R(\textbf{f})$, which is equivalent to regularized (or penalized) ML; here $\beta$ is a parameter which determines the weight of the prior. In PET and SPECT, several formulations of $R(\textbf{f})$ have been proposed by assuming that the images are piece-wise smooth. Using specific edge-preserving priors and appropriate $\beta$ values, image noise can be effectively reduced without loss of resolution. In spite of the great potential of Bayesian reconstruction, only few works report the use of priors for Compton-camera data [105, 108–110].

**Fig. 6:**
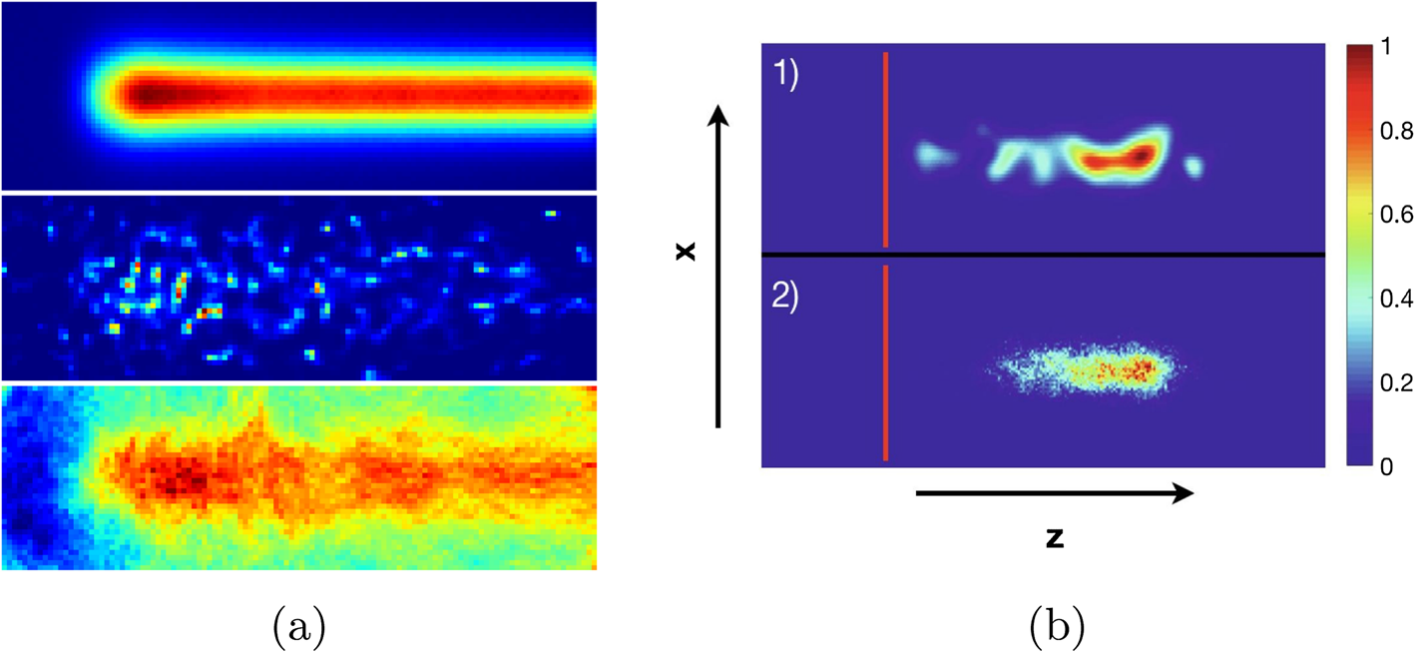
Examples of the different behavior of MLEM and OE in presence of strong statistical fluctuations (adapted from [111] (Images licensed under CC BY 4.0.). **a** From top to bottom: Original prompt-gamma distribution (simulation); MLEM reconstruction, and OE reconstruction. **b** Reconstructed prompt-gamma emission for a 150 MeV proton pencil beam incident from the left on a water phantom measured with the Polaris-J Compton Camera, reprinted from [112] (Image reproduced with permission of Elsevier)

Another perspective to the reconstruction problem is offered by the Origin Ensemble (OE) algorithm. OE was initially proposed for emission tomography data [113] but later extended to Compton-camera imaging [114]. Since then, OE and its variations have won great popularity for CC imaging, see, e.g., [104, 111, 112, 115]. Compared to ML-based approaches, OE does not require forward and backward projections, but all operations are carried out in the image domain. The reconstructed image is obtained by averaging over possible realizations of the unknown image, which are obtained by means of the Metropolis-Hasting algorithm. In this way, OE not only provides an estimate of the expected emission distribution, but also of the variance affecting each image voxel. Similarly to MLEM, OSEM and MAP approaches, OE requires a model of the system matrix and the sensitivity, and it also allows for compensation of the limited energy resolution and spatial uncertainties [104, 116]. OE is also very sensitive to data fluctuations and discretization of the field of view, although the effects on the image are different as for MLEM [111, 112]. An example of how both algorithms cope with extremely low data is shown in Fig. 6. A comparison between the two sets is not meaningful since the cameras, the data and the reconstruction parameters are different (e.g., the algorithms were let to convergence in Fig. 6a while early stopping was used for Fig. 6b).

Although OE relies on a Bayesian interpretation of the imaging process, regularization by means of priors has been only attempted for PET using the truncated flat and the conjugate priors [117]. More recently, other priors have been successfully adapted for OE [118]; for an appropriate choice of the regularization parameter, the image quality can be notably improved. Alternatively, noise could be reduced by introducing some intermediate smoothing steps into the OE algorithm [119].

Equation 2 ignores the effects of attenuation and scatter in the patient, or accidental coincidences (randoms). In PET, attenuation is partly compensated within MLEM and its variations by modifying the system matrix (and subsequently the sensitivity) with a correction term which only depends on *i*. Scatter and accidental coincidences can be taken into account in the forward-projection step, i.e., the denominator is modified to $y_i^{(k)} + \bar{n}_i + \bar{r}_i$, where the latter two terms correspond to the estimated contribution of scatter and randoms to the detection element *i*, respectively. Although this methodology could be extended to CC imaging, this has been seldom attempted; a possible reason is that current Compton cameras are not operated in conjunction with CT or MRI scanners, in contrast to SPECT/CT, PET/CT or PET/MRI systems. These additional scans would support the calculation of the attenuation and scatter contributions. Instead, more practical but less accurate procedures have been implemented [120, 121].

**Fig. 7:**
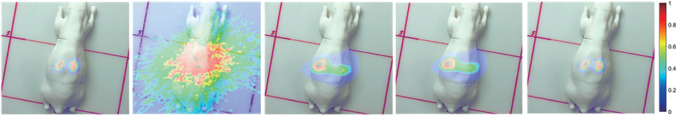
Reconstructed data of a 3D-printed mouse phantom with a brain-like volume filled ${}^{18}$F-NaF and acquired with a CZT-based CC, overlapped onto a photograph of the phantom. From left to right: Exact activity distribution, Monte-Carlo backprojection method, LM-MLEM with PSF modeling, modified OE with subsets and PSF modeling, and MCBP-CCnet, an algorithm that combines Monte-Carlo sampling-based backprojection with a dedicated convolutional neural network. Figure extracted from [122] (Figure reproduced with permission of John Wiley and Sons)

The recent rise of deep learning (DL) has also found resonance in the field of image reconstruction for Compton cameras. Promising results have been obtained for point sources [123] and small hot lesions in a cold background [122], see Fig. 7. It still remains open how good DL-based reconstruction will perform for extended radioactive sources.

### Reconstruction of the interaction sequence and event selection

An issue which is common to Compton cameras, independently of their application, is the accurate determination of the interaction sequence. For the energies considered in medical applications, Compton scatter is mainly forward peaked, but backscatter is not precluded. Angles smaller than 90 degrees can also lead to ambiguities, in particular for extended activity distributions with photons impinging onto the camera in oblique angles. Due to the limited energy, position and timing resolution of current detectors, the restrictions imposed by Compton kinematics on the scattering angle might not suffice to identify the first interaction. This fact has led to the development of a variety of sequence reconstruction algorithms, from analytical approaches [124] to more sophisticated methods based on artificial neural networks [125].

Within the frame of particle therapy, event selection instead of compensation during the reconstruction is often attempted to reduce the impact of accidental coincidences and fortuitous events [69], and/or to compensate for the wrong estimation of the unknown initial energy [126]. Machine learning, in particular deep neural networks, has also shown some potential in this regard [127–130].

## Hybrid PET/Compton camera systems

**Fig. 8:**
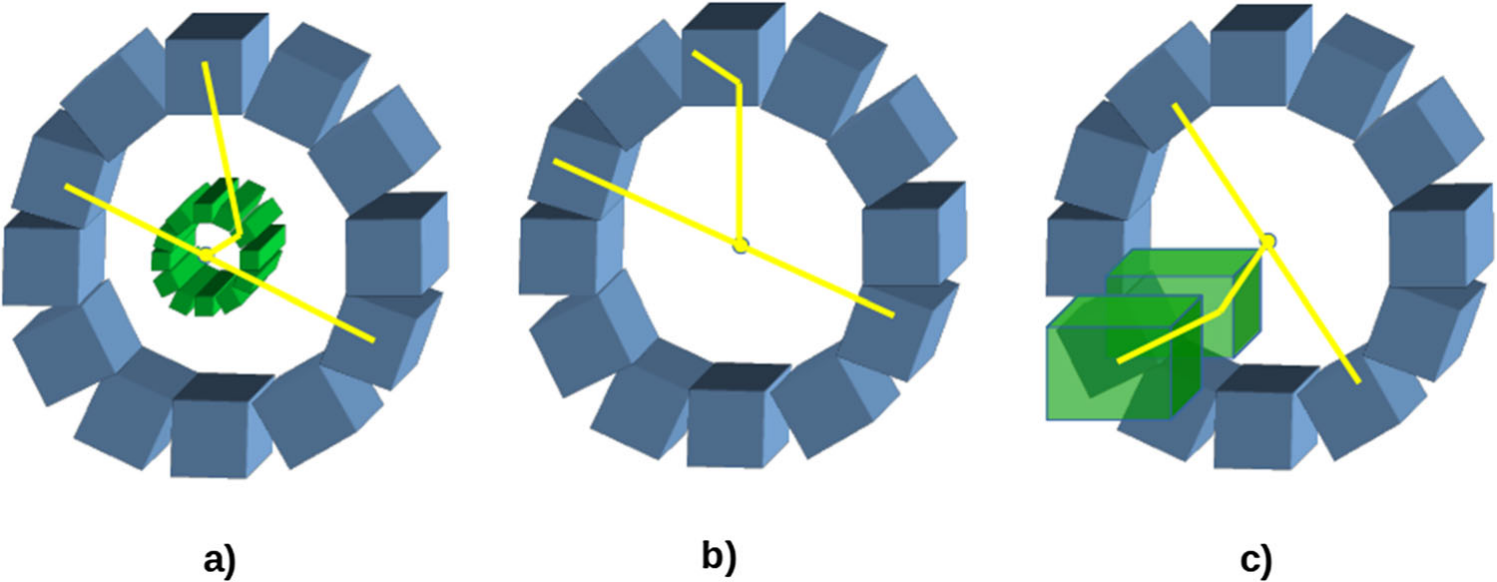
Possible configurations of combined PET/CC systems. **a** Inner scatter detector inside a conventional PET ring. **b** Events scattered in the ring detectors are employed for Compton imaging. **c** The Compton camera is located outside the PET ring to avoid interference

Commercial bimodal systems have demonstrated the added value that emerges from combining two modalities in a single system. In the same vein, the combination of PET and CC imaging could cover application areas in which each of these modalities alone has some limitations. However, to achieve this, the system design and operation should follow the requirements to obtain a good performance with both modalities independently. Possible implementations of a detector for these two modalities combined are represented schematically in Fig. 8. Figure 8a represents a double-ring configuration in which the inner ring, the scatter detector, can be a partial ring. The outer ring is the second detector for the Compton camera and both rings can act as PET detectors. In Fig. 8b, a single detector ring is employed in which different interactions can be distinguished. In configuration Fig. 8a, the PET signal can be negatively affected by the presence of the Compton detectors within the PET FOV. To avoid this effect, other geometries have been proposed placing an independent Compton camera beside the PET ring, such as in Fig. 8c.

In Compton cameras, the scatter detector should be placed as close as possible to the source in order to maximize both sensitivity and spatial resolution. The position of the second detector will also affect those two parameters, but inversely: a larger distance between the two detectors improves the resolution and reduces the sensitivity [131]. Configurations in Fig. 8a, c can adapt the geometry to optimize both parameters, while in configuration Fig. 8b the geometry is less flexible in this sense. The combination of these two imaging modalities can achieve good results in those applications in which the performance of the Compton cameras can be optimized, e.g., those in which the scatter detector can be placed close to the image region as in small animal or dedicated scanners such as brain scanners. Simultaneous PET and CC imaging has also been proposed for hadron therapy treatment monitoring.

Different working modes are possible depending on the isotopes employed. Images in PET and Compton modalities can be obtained acquiring data with positron or single photon emitting radiotracers independently for each modality, or also imaging single 511-keV photons detected in Compton mode.

The $\beta ^{+}$ decay of some PET radioisotopes also results in the emission of photons. While traditionally this has been something to be avoided or that might be tolerated [132], the use of such isotopes allows detection of a single isotope in both imaging modalities, i.e., the two annihilation photons plus the single gamma. When the gamma emission takes place quasi-simultaneously (typically a few ps later) to the positron emission, systems have been proposed to detect $\beta ^{+}$-$\gamma$ coincidences. Simultaneous detection of annihilation and deexcitation photons has been achieved on the first experiment of positronium detection carried out with the total body PET scanner J-PET [133]. Also, three-gamma interactions have been observed in the search for orthopositronium decay events [134]. J-PET is made of 192 plastic scintillator strips arranged into three concentric layers. Compton interactions of the photons in the detectors are registered and data are collected in continuous, triggerless, readout mode.

**Fig. 9:**
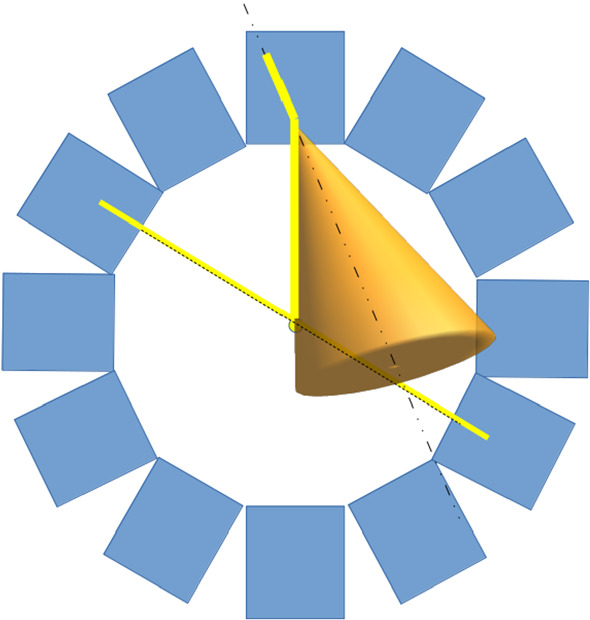
Three-gamma or $\beta ^{+}$-$\gamma$ imaging. The intersection of the Compton cone with the PET LOR determines the photon origin, reducing the image background

When CCs are employed in combination with PET scanners, the origin of each event can be constrained to the intersection between the line defined by the coincidence detection of two 511-keV photons and the cone surface related to the detection of the single gamma and calculated using Compton kinematics [135, 136] (Fig. 9).

A thorough review of the isotopes suitable for $\beta ^{+}$-$\gamma$ imaging can be found in [137, 138]. Optimum candidates should undergo $\beta ^{+}$-$\gamma$ decay followed by the emission of a photon of several hundred keV to 1 MeV, emitted within picoseconds and, ideally, with no additional photon emissions to avoid background. Half-lives from a few hours to a few days and feasible production are also essential requisites for their clinical use. Some of them have the additional advantage to form theranostic pairs with other isotopes. Among the most promising candidates are ${}^{44m}$Sc, ${}^{48}$V, ${}^{86}$Y, ${}^{94}$Tc, ${}^{152}$Tb,${}^{34m}$Cl, or the ones already popular in nuclear medicine in ${}^{66}$Ga, ${}^{72}$As, ${}^{82}$Sr/${}^{82m}$Rb or ${}^{124}$I. The very short-lived isotopes ${}^{10}$C and ${}^{14}$O, which have been proposed for online PET monitoring for hadron therapy, are also $\gamma$ emitters, thus being candidates for this modality. ${}^{22}$Na is also a $\beta ^{+}$-$\gamma$ emitter generally employed in laboratory tests due to its long half-life. Another interesting candidate for simultaneous PET/CC imaging is ${}^{89}$Zr, which emits a 909 keV photon uncorrelated to the $\beta ^{+}$ emission.

### PET/CC studies and developments

The use of a silicon scatterer inside a conventional PET ring was first tested in [36]. The system was composed of two silicon pad detectors placed at 17 cm from each other and four BGO crystal blocks from a PET scanner, each pair placed at both sides of the silicon detectors. The study showed sub-millimeter resolution (980 $\mu$m FWHM) at the center of the FOV in PET employing the silicon events and suggested the possibility of combining both modalities.

Monte-Carlo simulation studies have explored the $\beta ^{+}$-$\gamma$ imaging technique with different types of detectors. In [139], a LXe time projection chamber (TPC) as a Compton camera was simulated in GEANT3 with the aim of locating a ${}^{44}$Sc source in three dimensions with a few mm resolution, using $\beta ^{+}$-$\gamma$ coincidences. The Compton camera was placed in one of the sides of an existing micro-PET system, similarly to configuration c in Fig. 8, at 10 cm from the PET center. The micro-PET consisted of a 26 cm diameter ring of LSO crystals with a field of view of 7.6 cm; the source was located at the PET center. With a Compton camera energy resolution of 4.3$\%$ FWHM at 1.157 MeV and 5.7$\%$ at 511 keV, an angular resolution of $\sigma =1.25^{\textrm{o}}$ was obtained, which led to a spatial resolution of 2.3 mm at the source location. The overall system (micro-PET+Compton telescope) sensitivity to $\beta ^{+}$-$\gamma$ is 0.14$\%$. Experimental data with the XEMIS1 prototype showed the feasibility of a LXeTPC as a Compton camera. XEMIS1 had an active volume of 2.8 cm $\times$ 2.8 cm $\times$ 12/6 cm. It was tested with ${}^{22}$Na, obtaining an energy resolution of 5$\%$ at 511 keV. XEMIS2 was designed as a monolithic cylindrical camera to contain up to nearly 200 kg of LXe, composed of two identical back-to-back LXeTPCs, with an active volume of 12 cm drift length each, inner radius of 7 cm and outer radius of 19 cm [140].

This technique was also explored in [141] in a simulation work employing MEGAlib. The system consisted of four Compton camera modules in a quadratic arrangement. Each module was composed of a double-sided silicon strip detector with an active area of $50 \times 50$ mm$^2$ and 2 mm thickness as scatterer, placed at 3.5 cm from the center of the system, and a LaBr$_3$ scintillator crystal of size $50 \times 50 \times 30\,\textrm{mm}^3$ as second detector. Two ${}^{22}$Na point-like sources separated by 0.4 mm were placed at the center, inside an H$_2$O sphere of 6 cm diameter. The two sources could be clearly be resolved, and a spatial resolution of 0.2 mm FWHM in each direction was obtained. The coincidence detection efficiency was $1.92\times 10^{-7}$ per ${}^{22}$Na decay. The intersection between the LOR and the Compton cone could be identified in 25$\%$ of the events. For a source activity of 0.7 MBq, much lower than that employed in conventional PET, an exposure time of 450 s would result in 20 intersections, which are sufficient for source reconstruction. An efficiency comparison with standard (non-TOF) PET was carried out in [136]. While the sensitivity in terms of number of intersections required to localize a point source is higher, it falls behind when considering the reconstruction efficiencies, which should be optimized. The technique was also proposed for imaging ${}^{10}$C or ${}^{14}$O in hadron beam irradiation.

**Fig. 10:**
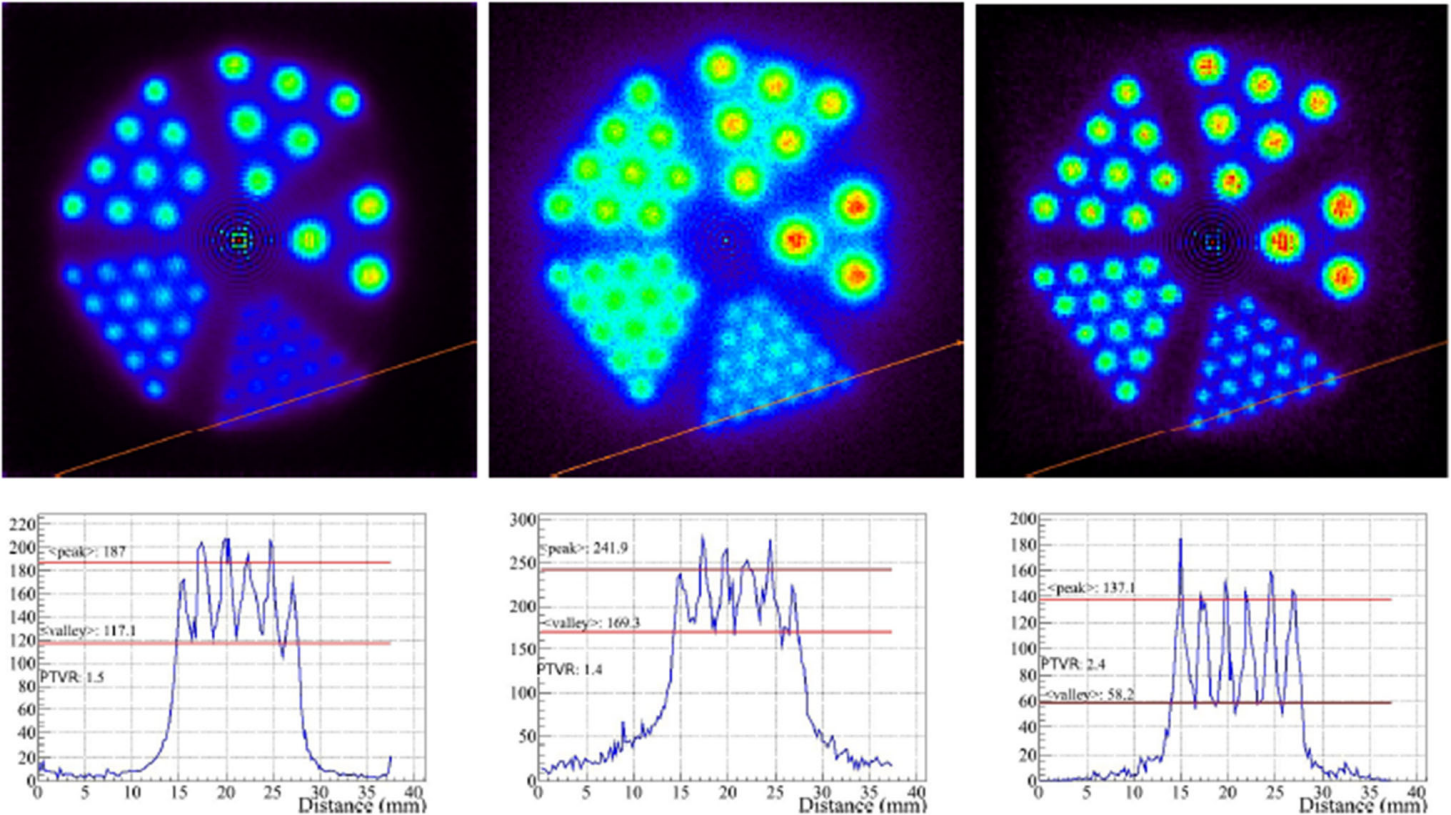
Images of a Derenzo phantom with rods ranging from 1.2 to 3 mm diameter, obtained from three-gamma events generated by realistic simulations and reconstructed with LM-OSEM. Left, LM-OSEM on entire LORs. Middle, LOR-cone intersections. Right, LM-OSEM on LOR segments of ± 5 mm around LOR-cone intersections. Image extracted from [143] ($\textcircled {c}$ IOP Publishing Ltd and Sissa Medialab srl. Reproduced by permission of IOP Publishing. All rights reserved)

The Monte-Carlo simulation study presented in [142] evaluated the three-gamma imaging technique with the insertion of a CZT inner ring in the ClearPET small animal scanner, made of LYSO-LuYAP scintillator crystals. Two cases were simulated, with 295 mm and 135 mm diameter. In both cases, the CZT ring diameter was 50 mm. Images were obtained with simulated ${}^{44}$Sc, ${}^{48}$V and ${}^{22}$Na point and 1 mm diameter sources. The results showed an improvement of the spatial resolution with the three-gamma system with respect to the PET-only system employing three-gamma events. However, the number of valid events was significantly reduced since the coincidence of four interactions (two for PET and two for Compton) is required. The simulation study was followed in [143], to compare the results with those of conventional PET. In this case, the system consisted of two concentric CdTe detector rings separated by 10 cm. The inner ring had 5 cm inner radius, 20 mm thickness and 25 cm axial length, as the outer ring. A Derenzo phantom containing ${}^{22}$Na filled rods with radii ranging from 0.51 to 1.5 mm was simulated and images reconstructed with LM-OSEM. While the three-gamma technique showed noise reduction in the images, conventional two-gamma PET interactions yielded better images due to superior statistics. However, the two modalities were not combined. Figure 10 shows the images of a Derenzo phantom with rods ranging from 1.2 to 3 mm diameter, obtained from three-gamma events generated by realistic simulations and reconstructed with LM-OSEM.

More recently, a so-called *whole gamma imaging* (WGI) system was assembled and tested [144]. The system, made of a scatter ring of 20 cm diameter inserted in a PET ring of 66 cm diameter was able to obtain both PET and Compton images and also work in three-gamma mode. The PET detector consisted of four rings of 40 modules composed of pixelated GSOZ crystals stacked in four layers and coupled to position sensitive PMTs. The scatter detector was composed of two rings of 20 modules each, with a single layer of GAGG crystals coupled to SiPMs. ${}^{137}$Cs and ${}^{22}$Na point sources and a vial filled with ${}^{44}$Sc were employed in the imaging tests. The sensitivity and the spatial resolution of the single gamma mode were 0.22$\%$ and 4.4 mm FWHM respectively, measured with the ${}^{137}$Cs source at the 8 cm off-center position, and 13.1 mm FWHM at the center position. In PET modality, employing only events from the outer ring, the spatial resolution obtained with the ${}^{22}$Na point source was about 2 mm FWHM at all positions tested, with a sensitivity much higher (about 1.5$\%$) than in Compton mode. Three-gamma imaging was tested with both ${}^{22}$Na and ${}^{44}$Sc sources. The average spatial resolutions were 4.9 mm and 6.7 mm, respectively. The sensitivity was about 1/3000 compared to that of the PET mode. In the three-gamma mode, images were obtained just by plotting the intersecting points between each LOR and Compton cone of the same event and no image reconstruction was applied. Further improvements in the system, including the development of appropriate image reconstruction codes, are necessary to demonstrate the potential of WGI.

**Fig. 11:**
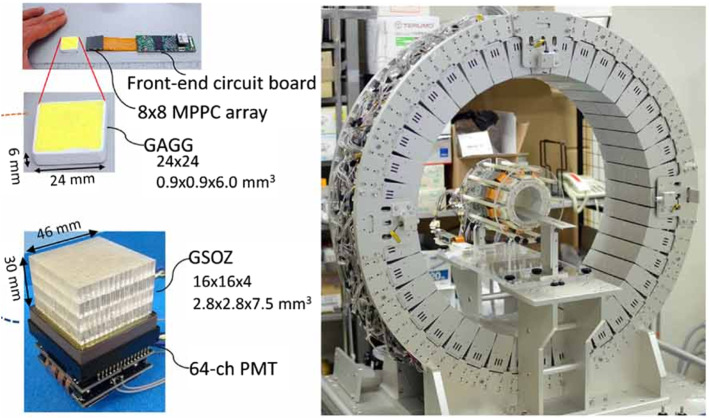
Components and photograph of the WGI prototype, with a 94 mm diameter inner ring of GAGG crystals and a 660 mm diameter outer ring of GSOZ crystals. From [121] (Image reproduced under the terms of the Creative Commons Attribution 4.0 license, $\textcircled {c}$ Institute of Physics and Engineering in Medicine. Reproduced by permission of IOP Publishing Ltd. All rights reserved)

**Fig. 12:**
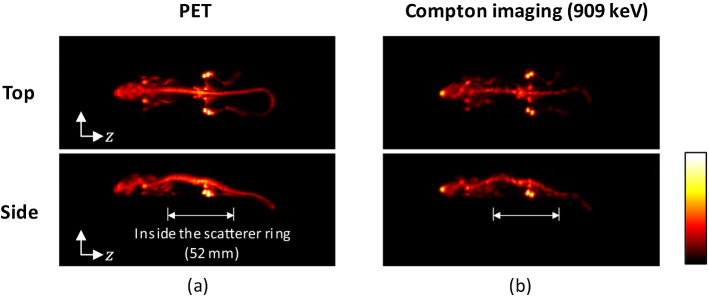
Maximum-intensity-projection images (top and side views) of a mouse administered ${}^{89}$Zr-oxalate using the Whole-Gamma-Imaging prototype: **a** Only PET data, and **b** only Compton data. Image from [121] (Image reproduced under the terms of the Creative Commons Attribution 4.0 license, $\textcircled {c}$ Institute of Physics and Engineering in Medicine. Reproduced by permission of IOP Publishing Ltd. All rights reserved)

The system was also tested on a mouse using a reduced scatter ring of 94 mm diameter (shown in Fig. 11) and employing ${}^{89}$Zr [121]. Tests with a phantom showed that WGI achieved spatial resolution better than 3.0 mm at the peripheral region with lower resolution at the center, while PET resolved 2.2 mm rods clearly at any location. The mouse bony structures could be clearly appreciated in PET images and Compton images were in agreement (Fig. 12 ). Efforts are addressed to enhance the energy resolution of the scatted detector in order to improve Compton spatial resolution.

The combination of PET and Compton imaging modalities was also assessed experimentally in [145]. The system employed consisted of two detector modules placed at 180$^\circ$. Each of them was composed of two layers of pixelated GAGG crystals coupled to SiPMs separated 30 mm. The system was tested both in PET and Compton modes independently with different sources, and also in both modalities simultaneously rotating the PET system to acquire tomographic images. Two 10-ml syringes filled with ${}^{18}$F-FDG and with ${}^{111}$InCl$_3$ were placed at the center. Images were reconstructed employing MLEM and FBP for PET and Compton respectively. Although the spatial resolution in Compton mode was worse that in PET, successful location of the reconstructed sources in both modalities was achieved, with the PET image showing only the ${}^{18}$F-FDG source, and the Compton image being able to image both the 245 keV photons from ${}^{111}$In and the 511-keV photons from ${}^{18}$F-FDG.

**Fig. 13:**
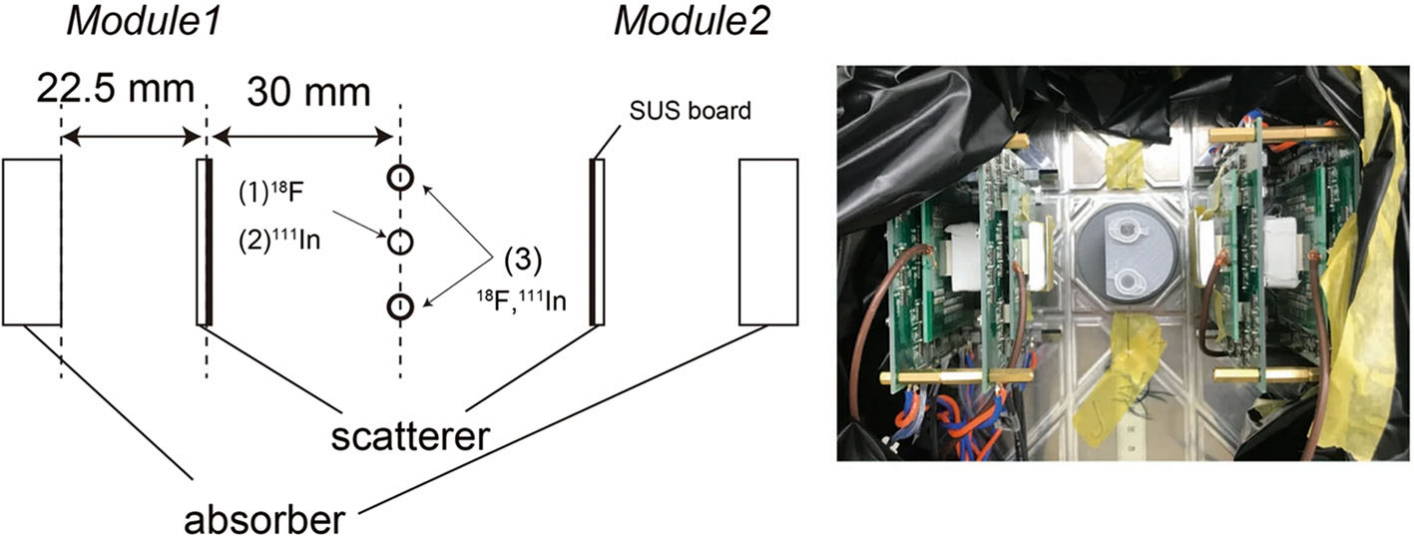
Experimental setup for simultaneous PET and Compton imaging, composed of two modules in a rotating stage, each one consisting of two HR-GAGG crystal arrays coupled to SiPMs. (From [146] (Article [146] licensed under a Creative Commons Attribution 4.0 International License, http://creativecommons.org/licenses/by/4.0/))

Simultaneous in vivo imaging combining PET and SPECT of a tumor-bearing mouse was achieved in [146]. A system composed of two modules attached to a rotating stage was employed (see Fig. 13). Each module was composed of two HR-GAGG crystal arrays coupled to SiPMs. The inter-detector distance was 22.5 mm, and the distance from the first detector to the source was 30 mm. The mouse was injected with ${}^{18}$F-FDG and an ${}^{111}$In-labeled ligand. Compton images were reconstructed with MLEM, and PET images with a plain backprojection algorithm. In the Compton image, the 245 keV of the ${}^{111}$In-antibody could be visualized strongly in the tumor and also in the liver. In the PET image, ${}^{18}$F-FDG uptake was observed in several organs including bladder, heart and brown adipocytes. The Compton image of 511-keV photons of ${}^{18}$F-FDG also showed accumulation in the bladder, which was not visible in the PET image due to its limited FOV. None of the ${}^{18}$F-FDG images showed accumulation in the tumor due to low uptake. A full ring is under development.

Another experimental verification following the work in [136, 141] was recently carried out in [147] with a system composed of two PET detector heads placed at 100 mm from each other and a two-layer CC placed perpendicularly to the line connecting the two PET detectors, at 55 mm distance. The four detectors were made of pixellated crystals coupled to SiPM arrays. The CC scatterer and the PET heads were made of 16 $\times$ 16 GAGG crystals of 1.45 $\times$ 1.45 $\times$6 mm$^3$ size, with an energy resolution of 10.4±0.2$\%$ at 662 keV. The absorber consisted of a three-layered, staggered LYSO block built from 1.2 $\times$ 1.2 $\times$ 6.66 mm$^3$ crystals. The distance between the scatter and the absorber was 43 mm. The ASIC TOFPET v2c was employed for readout. Synthetic triple-coincidence events from a point-like source placed off-center, at 50 mm from the CC, were obtained by combining in the post-processing events from a single measurement, but triggered in PET-only and Compton-only mode. Via event-wise intersection of the line-of-response and the Compton cone, a 3D image of the source was obtained with only 77 events, with a spatial resolution similar to the one obtained in PET-only mode (3.9 mm and 3.3 mm in the two dimensions in a plane parallel to the PET detectors, and 12.9 mm along the line connecting the PET heads). The system needs to be upgraded to improve the overall geometrical efficiency and obtain sufficient data in three-gamma mode.

The system proposed in [148] aims to visualize the spatial distribution of non-conventional positron emitters inside small aquatic animals. It consists of a LYSO ring of 28 mm radius together with a three-layer LaBr$_3$ Compton camera in lateral configuration, see Fig. 8c; the latter is inspired in the MACACO system [131], whereas the PET part is an extension of the current MERMAID PET prototype [149]. These first studies with ${}^{89}$Zr and ${}^{124}$I have been carried out employing *CCMod*, the GATE module for Compton-camera simulation [150].

### Image reconstruction of hybrid PET/CC

**Fig. 14:**
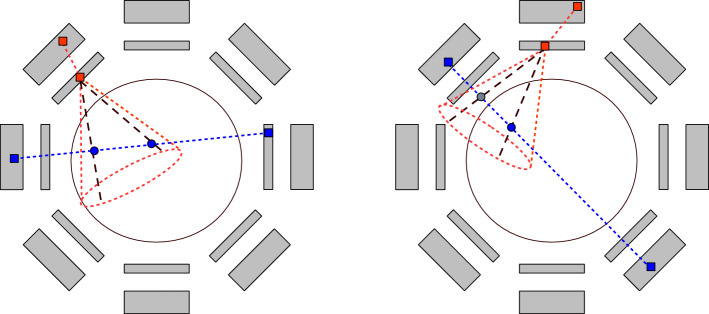
Exemplary illustration of the COR (dashed lines, orange) and the LOR (blue line) of a true three-gamma event, as well as the corresponding intersection between the two (blue circles). For a true event under ideal conditions, either two intersection points (left) or only one (right) fall within the FOV

To fully exploit the capabilities of hybrid PET/CC systems, the reconstruction approach should be tailored to the particular combination of the two modalities and the type of measurement. For example, consider those systems aimed to detect $\beta ^{+}$-$\gamma$ coincidences, i.e., the two annihilation photons following a $\beta ^{+}$-decay and a single gamma photon. Under ideal conditions, the image reconstruction process could be simplified to histogramming the intersection points between the COR and LOR of each three-gamma event (*LOR-Cone intersection*, LCI). If the FOV is limited or the extension of the object is known, either one or two intersection points can be found per true event (see Fig. 14). In the latter case, only one of the two points corresponds to the true emission, so that the second one contributes to increase the image noise.

Measurement uncertainties can be taken into account using only a segment of the LOR, the one which results from intersecting an ideal LOR and the VOR related to the Compton event [151]. Next, a simple model based on ray-tracing could be further used to compute $a_{ij}$ as the geometrical contribution of the LOR segment to a certain voxel *j*. In the same way as TOF information is included into PET reconstruction, LM-MLEM can be adapted to three-gamma PET using a Gaussian kernel with weights $w_{ij}$ centered at the LCI [152]. In any case, the uncertainty related to positron range remains.

Another reconstruction concept is required for non-correlated measurements of Compton events and PET events. The most straightforward approach is to independently reconstruct the two detection channels using two independent reconstruction algorithms, one for Compton camera data, and one for PET data (see Fig. 12). This concept can be further extended with a third channel for triple-gamma imaging [144]. Given that the various types of measured data originate from the same radiotracer distribution, synergistic approaches able to reconstruct all events within a single framework are more appropriate. Following this idea, a practical extension of LM-OSEM (see Eq. 3) has been recently introduced in [153] and can be expressed as follows:

5 $$\begin{aligned} \begin{aligned} f_j^{(k, l+1)}&= \alpha \frac{f_j^{(k, l)}}{s^{{\rm PET}}_j} \sum _{i\in N^{{\rm PET}}_l} \frac{1}{\sum _{m=1}^J a^{{\rm PET}}_{im} f_m^{(k)} } a^{{\rm PET}}_{ij}\\&\quad + (1- \alpha ) \frac{f_j^{(k, l)}}{s^{{\rm CC}}_j} \sum _{i\in N^{{\rm CC}}_l} \frac{1}{\sum _{m=1}^J a^{{\rm CC}}_{im} f_m^{(k)} } a^{{\rm CC}}_{ij}, \end{aligned} \end{aligned}$$

where $\alpha$ weights the relative contributions of the two modalities; within one sub-iteration, the data are handled separately using dedicated sensitivities, $\mathbf {S^{\rm PET}}$ and $\mathbf {S^{\rm CC}}$, as well as system matrices, $\mathbf {A^{\rm PET}}$ and $\mathbf {A^{\rm CC}}$. This approach assumes that, within an iteration, the differences in the contribution of the two modalities can be reflected by one single parameter and that the same voxel size is appropriate for both PET and Compton camera. A joint reconstruction scheme based on MLEM was suggested for Compton events within PET; it was derived from considering the likelihood function as the sum of the contributions of the individual event types [102, 154]. Unified frameworks might be problematic because the spatial resolution of Compton-camera data is usually worse than for PET data. This issue was tackled in [155] within the context of a high-resolution PET system based on CZT detectors, where three different joint reconstruction frameworks were briefly presented. Unfortunately, the results were not conclusive. In any case, to fully exploit hybrid PET/CC imaging, further developments in image reconstruction and system modeling will be desirable.

## Conclusions

In the last decade, new technological advances have led to re-thinking of Compton cameras as an imaging modality with great potential for medical applications. In conventional clinical settings, state-of-the-art Compton cameras cannot compete with well-established methods such as PET and SPECT. However, there are specific scenarios in which Compton cameras are an advantageous alternative, even more if they are combined with PET, either through a single detector concept or by embedding two devices into one system. To date, a direct comparison between modalities is difficult as most Compton-camera prototypes are often limited by their size, and image reconstruction and degradation correction methods are not yet as advanced as for PET or SPECT.

Compton cameras can offer advantages over gamma cameras in some aspects since they are well suited for multitracer imaging, which is crucial when the physiological functions studied depend on each other (e.g., perfusion and metabolism), and for imaging high-energy radiotracers, of increasing use, for example, in radionuclide therapy. Their potential benefits are being demonstrated in experimental tests.

The combination of PET and Compton imaging can benefit from the improved resolution and sensitivity of current PET technology and, at the same time, overcome PET limitation in the use of multiple radiotracers. Such a system can provide simultaneous assessment of different radiotracers under identical conditions and reduce errors associated with physical factors that can change between acquisitions. Moreover, additional imaging modalities can be exploited, such as three-gamma or $\beta ^{+}$-$\gamma$ imaging, which can provide excellent image resolution. Those events, although scarce, can be combined with PET or Compton events to improve system resolution.

This field is experiencing fast progress and achieving promising results that might soon show their benefits in clinical scenarios. To this end, further progress is being made in the field-of-image reconstruction, correction of degradation phenomena and design optimization. Equally important is the development of high-performance, cost-effective solutions.

## Data Availability

With the exception of the images shown in Fig. 5, this review article refers exclusively to the results of the publications listed below and their references. New data are not associated with the manuscript.
